# Extracellular matrix degradation pathways and fatty acid metabolism regulate distinct pulmonary vascular cell types in pulmonary arterial hypertension

**DOI:** 10.1177/2045894021996190

**Published:** 2021-03-02

**Authors:** Sharon Mumby, F. Perros, C. Hui, B.L. Xu, W. Xu, V Elyasigomari, A. Hautefort, G. Manaud, M. Humbert, K.F. Chung, S.J. Wort, I.M. Adcock

**Affiliations:** 1 Respiratory Science, NHLI, Imperial College London, London, UK; 2 UMRS 999, Laboratoire d’Excellence en Recherche sur le Médicament et l’Innovation Thérapeutique, INSERM and Paris-Sud, Le Plessis Robinson, France; 3 Centre de Recherche de l’Institut Universitaire de Cardiologie et de Pneumologie de Québec, Laval University, Québec, Canada; 4 Centre for Respiratory & Critical Care Medicine, The University of Hong Kong-Shenzhen Hospital, Shenzhen, China; 5 Department of Computing, Data Science Institute, Imperial College London, London, UK; 6 Département Hospitalo-Universitaire Thorax Innovation, Centre de Référence de l'Hypertension Pulmonaire Sévère, Service de Pneumologie et Réanimation Respiratoire, Hôpital de Bicêtre, Le Kremlin-Bicêtre, France; 7 National Pulmonary Hypertension Service, Royal Brompton Hospital, London, UK

**Keywords:** endothelial cell dysfunction, pulmonary artery smooth muscle cells, RNA-sequencing

## Abstract

Pulmonary arterial hypertension describes a group of diseases characterised by raised pulmonary vascular resistance, resulting from vascular remodelling in the pre-capillary resistance arterioles. Left untreated, patients die from right heart failure. Pulmonary vascular remodelling involves all cell types but to date the precise roles of the different cells is unknown. This study investigated differences in basal gene expression between pulmonary arterial hypertension and controls using both human pulmonary microvascular endothelial cells and human pulmonary artery smooth muscle cells. Human pulmonary microvascular endothelial cells and human pulmonary artery smooth muscle cells from pulmonary arterial hypertension patients and controls were cultured to confluence, harvested and RNA extracted. Whole genome sequencing was performed and after transcript quantification and normalisation, we examined differentially expressed genes and applied gene set enrichment analysis to the differentially expressed genes to identify putative activated pathways. Human pulmonary microvascular endothelial cells displayed 1008 significant (*p* ≤ 0.0001) differentially expressed genes in pulmonary arterial hypertension samples compared to controls. In human pulmonary artery smooth muscle cells, there were 229 significant (*p* ≤ 0.0001) differentially expressed genes between pulmonary arterial hypertension and controls. Pathway analysis revealed distinctive differences: human pulmonary microvascular endothelial cells display down-regulation of extracellular matrix organisation, collagen formation and biosynthesis, focal- and cell-adhesion molecules suggesting severe endothelial barrier dysfunction and vascular permeability in pulmonary arterial hypertension pathogenesis. In contrast, pathways in human pulmonary artery smooth muscle cells were mainly up-regulated, including those for fatty acid metabolism, biosynthesis of unsaturated fatty acids, cell–cell and adherens junction interactions suggesting a more energy-driven proliferative phenotype. This suggests that the two cell types play different mechanistic roles in pulmonary arterial hypertension pathogenesis and further studies are required to fully elucidate the role each plays and the interactions between these cell types in vascular remodelling in disease progression.

## Introduction

Pulmonary arterial hypertension (PAH) is a rare and incurable disease characterised by elevated mean pulmonary artery pressure (≥20 mmHg), increased vascular resistance and remodelling of the pulmonary vascular bed which ultimately leads to right heart failure and death.^1^ Despite improvements in PAH therapies, prognosis is poor with a survival rate of ∼60% three years after diagnosis.^2^ PAH is sub-classified into idiopathic (IPAH), heritable (HPAH), drug and toxin exposure and that associated with various conditions including connective tissue and congenital heart disease.^1^ Although there is variable aetiology, all cases of PAH show a similar histological appearance with characteristic remodelling of the pulmonary arterioles including plexiform lesions in severe cases.^3^ Genetic mutations, in particular those related to bone morphogenetic protein receptor (BMPR)-II and related proteins, are found in most cases of HPAH and a proportion of IPAH.^4,5^ PAH patients who do not have mutations in BMPR-II still show a reduced expression and function of this receptor and associated signalling molecules.^6^ Furthermore, the penetrance is only about 20%, indicating that further “hits” are necessary for disease progression. Less common mutations have also been discovered such as SOX-17, AQP and KCNK3.^7–9^

The precise processes underpinning the vascular remodelling seen in PAH remain unknown but include vasoconstriction, proliferation of intimal, medial and adventitial cells, thrombosis and inflammation.^10,11^ The primary trigger, which may or may not involve a genetic pre-disposition as described above, is likely to involve the endothelial cell (EC). This cell type is directly exposed to changes in blood flow, oxygenation and circulating factors including cytokines, hormones and drugs. It is probable that early changes include increased apoptosis and disruption of the endothelial layer resulting in exposure of the smooth muscle layer to soluble growth factors and cytokines.^10,11^

If injury is sustained, then phenotypic changes occur in the endothelial layer resulting in the release of additional mediators into the vascular environment which can promote survival of surrounding ECs leading to dysregulated proliferation and aberrant neoangiogenesis.^12^ Mediators released by ECs also affect the underlying basement membrane and surrounding extracellular matrix (ECM) together with effects on smooth muscle cells and fibroblasts.^13^ Alterations in the composition and amounts of ECM contribute to the abnormal local environment in the remodelled pulmonary vessels of PAH patients.^14^ Thus, altered ECM can promote vascular remodelling in various ways. These include the release of large amounts of growth factors and other molecules held within the ECM, the release of fragments of ECM components known to modulate proliferation, migration and protease activation and exposure of functionally important sites in collagens, laminins or elastins.^14^ There is growing evidence showing that an imbalance between proteases (e.g. matrix metalloproteinases (MMPs), elastases and their inhibitors (tissue inhibitors of matrix metalloproteinases (TIMPs)) could occur in the pulmonary artery to promote vascular remodelling.^15–17^

In an attempt to unravel some of the cell-specific processes involved in the remodelling seen in PAH, we investigated differences in basal gene expression using RNA- and miRNA-sequence analysis between PAH and non-PAH controls using both human pulmonary artery smooth muscle cells (HPASMC) and pulmonary microvascular endothelial cells (HPMEC). A bioinformatics approach was utilised to identify the differentially expressed genes (DEGs) in each cell type and these genes were then subjected to pathway analysis. The number of DEGS in HPASMCs (229) were lower than those seen in HPMEC (1008) from PAH patients compared to controls. Pathway analysis revealed distinctive differences with HPMEC displaying down-regulation of ECM organisation and HPASMCs up-regulation of fatty acid metabolism suggesting a more energy-driven proliferative phenotype.

## Methods

### Tissue collection and cell isolation

#### Endothelial cells

Controls: Healthy HPMEC were purchased from Promocell GmbH (Heidelberg, Germany) and cultured according to the supplier’s instructions in Endothelial cell growth medium MV (Promocell GmbH, Heidelberg, Germany).

PAH: Lung parenchyma was collected at the time of lung transplantation from PAH patients, part of the French Network on Pulmonary Hypertension, a programme approved by our institutional Ethics Committee, and had given written informed consent (Protocol N8CO–08–003, ID RCB: 2008-A00485-50, approved on 18 June 2008).

To isolate HPMEC, a peripheral section of lung parenchyma was cut into 5-mm pieces and enzymatically digested using 2 U/mL dispase in hank's balanced salt solution (HBSS) for 90 min at 37°C. The sample was vortexed and flushed with a 10 mL pipette at 20-min intervals during digestion. The tissue homogenate was filtered using a 70 μm cell strainer (BD Falcon) and the filtrate was centrifuged for eight minutes, at 20°C, 500×*g*. The cell pellet was re-suspended and HPMEC were cultured in gelatin-coated (2 mg/mL) six-well plates in microvascular EC growth medium containing 6% growth supplement, 100 U/mL penicillin, 100 μg/mL streptomycin and 1.25 μg/mL fungizone. When the cells reached 90% confluence, immunomagnetic purification of HPMEC was performed using CD31 monoclonal antibody-labelled microbeads (Miltenyi Biotec) as previously described.^18^ Immunomagnetic purification of HPMEC was repeated three times at one-week intervals. Cells were then cultured under the same conditions and in the same media as the healthy ECs following the manufacturer instructions.

#### Smooth muscle cells

Controls: Lung parenchyma was collected from control subjects at lobectomy or pneumonectomy for suspected lung tumour. The study protocol was approved by the BRU Biobank at the Royal Brompton Hospital and Ethical approval was obtained for the study (#10/H0504/9), and all donors gave consent for the use of their tissue.

PAH: Lung parenchyma was collected at the time of lung transplantation from PAH patients, part of the French Network on Pulmonary Hypertension, a programme approved by our institutional Ethics Committee, and had given written informed consent (Protocol N8CO–08–003, ID RCB: 2008-A00485-50, approved on 18 June 2008).

Microvascular pulmonary arteries with a diameter smaller than 500 μm were dissected under a microscope from sections of lung parenchyma. To prevent any contamination by fibroblasts, adventitial tissue was removed from pulmonary arterial sections using forceps. HPASMC were isolated using the explant-outgrowth method previously described^19^ and cultured in Smooth muscle cell growth medium 2 (Promocell GmbH) as per the manufacturers recommendations. HPASMC upon culture displayed a ‘hills and valleys’ organisation typical of true smooth muscle cells and displayed alpha-smooth muscle actin (α)-SMA filaments and expressed desmin. Donor demographics are shown in Table 1. All HPMEC and HPASMC were used between passage 3 and 5.

**Table 1.** Donor demographics for the different cell types used in the study.

Donor	Sex (M/F)	Age (Years)	Cell type	Primary diagnosis	Co-morbidities
PAH	?	?	PASMC	IPAH	
PAH	F	42	PASMC	IPAH	
PAH	M	22	PASMC	IPAH	
PAH	F	32	PASMC	HPAH	
PAH	F	26	PMEC	HPAH	
PAH	F	45	PMEC	Pulmonary veno-occlusion disease (PVOD)	
PAH	M	53	PMEC	PVOD	
PAH	F	27	PMEC	IPAH	
Non-PAH	M	68	PASMC	Squamous cell carcinoma	CABG
Non-PAH	M	60	PASMC	Adenocarcinoma	Asthma
Non-PAH	F	40	PASMC	Non-small cell carcinoma	Breast cancer
Non-PAH	M	73	PASMC	Adenocarcinoma	Duodenal ulcer
Non-PAH	F	42	PMEC	N/A	N/A
Non-PAH	M	81	PMEC	N/A	N/A
Non-PAH	F	56	PMEC	N/A	N/A
Non-PAH	M	70	PMEC	N/A	N/A

PASMC: pulmonary artery smooth muscle cells; PMEC: pulmonary microvascular endothelial cells; CABG: coronary artery bypass grafts; PAH: pulmonary arterial hypertension; IPAH: idiopathic pulmonary arterial hypertension; HPAH: heritable pulmonary arterial hypertension.

### RNA isolation

Cells were grown to confluence under standard cell culture conditions and cells were harvested in RNA Later (RLT) buffer. RNA and miRNA were extracted at the same time using a miRNeasy kit (Qiagen Ltd, (Manchester, UK)). A nanodrop-lite (Thermo Scientific) was used to determine the RNA concentration.

### RNA and miRNA library construction and sequencing

Small RNA library construction was performed by the Beijing Genomics Institute (BGI, Shenzhen, China). Small RNAs (> 30 nt) were collected following gel separation and ligated to 3′ and 5′ adapters and reverse transcription (RT) was performed. The cDNA was expanded by 15 cycles of PCR, fragments size fractionated and gel purified before being used to generate DNA nanoballs (DNBs). DNBs were loaded into the patterned nanoarrays and single-end reads of 50 bp were read on the BGISEQ-500 platform (Shenzhen, China). Sequence data were analysed using the miRDeep module to obtain the read counts of known miRNAs. RNA was converted to cDNA using RT and adapters ligated. The products were purified and enriched using PCR amplification and DNBs generated. Stepwise sequencing was performed using the combinatorial probe-anchor ligation approach and read on the BGISEQ-500 system.

### Data analysis

After sequencing, the quality of the RNA-Seq and miRNA-Seq FastQC reads was assessed using Illumina HiSeq platform v0.11.5 (www.bioinformatics.babraham.ac.uk/projects/fastqc/). The raw reads were filtered to remove adaptor sequences, biological contaminations and low-quality reads and high-quality reads (>20 Phred score) were then mapped against the human reference genome (hg38) using STAR aligner version 2.5.3a.^20^ The average read-length for the RNA samples was 150 bp. The STAR quantMode option was set to GeneCounts to count the number of reads per gene to obtain the raw count matrix. An indexed genome similar to that for RNA-Seq was generated for the miRNA-Seq single-end reads. The reads were mapped against the created indexed genome using STAR and the count matrix corresponding to all miRNA-Seq samples were computed. Both count matrices from RNA-Seq and miRNA-Seq samples were imported to R (version 3.5.0) for downstream analysis.

Exploratory principle component analysis on the regularised log transformed data identified a tube effect that was subsequently accounted for. The raw counts for the RNA-seq and the miRNA-seq data were modelled using a generalised linear model for health and PAH. Differential expression analysis was performed using DESeq2 version 1.20.0,^21^ which is based on negative binomial testing. The default DESeq2 parameters were used to normalise the raw counts and estimate the dispersion and DEGs.

Enrichr (http://amp.pharm.mssm.edu/Enrichr/) was used for Pathway, Ontology and Transcription factor analysis of differentially regulated genes and microRNAs. All *p* values quoted are adjusted *p* values and LOG2 fold change is stated as fc in the text. Combined score was calculated by taking the LOG of the *p* value from the Fisher Exact test and multiplying that by the Z score of the deviation from the expected rank.

## Results

### DEGs in PAH-HPMECs compared to controls

HPMEC displayed 1008 DEGs which were significantly (*p* ≤ 0.0001) up- or down-regulated in PAH compared to controls, see Table 2A for the top 20 DEGs. Of these genes, 611 were still significant at *p* ≤ 0.000001. The top five most significantly changed genes, all of which were down-regulated with ≤ –3.3-fold change (fc) were *PKHD1L1, ITGA1, POSTN, RELN, COL4A5*. Of the significantly regulated genes, the top five most up-regulated were *CARD11, IFI27, ADAM15, IFI6, CCNA1* all with ≥3 fc except *ADAM15* with 0.7 fc, data not shown.

**Table 2.** The top 20 (A) significant (B) up-regulated and (C) down-regulated DEGs in human pulmonary microvascular endothelial cells from PAH patients compared to controls.

	log2 fold change	padj	Symbol
2A			
ENSG00000205038	–5.64942	7.87E-177	PKHD1L1
ENSG00000213949	–3.90634	5.01E-164	ITGA1
ENSG00000133110	–3.57053	4.47E-137	POSTN
ENSG00000189056	–3.86078	4.65E-135	RELN
ENSG00000188153	–3.34501	2.72E-119	COL4A5
ENSG00000115380	–1.30613	2.73E-116	EFEMP1
ENSG00000138722	–1.35321	1.70E-111	MMRN1
ENSG00000203875	–2.43038	5.74E-107	SNHG5
ENSG00000170421	–3.55254	9.14E-99	KRT8
ENSG00000174175	–3.67888	6.00E-80	SELP
ENSG00000162706	–7.47537	4.69E-78	CADM3
ENSG00000143341	–2.77443	2.07E-77	HMCN1
ENSG00000137573	–3.98658	7.29E-77	SULF1
ENSG00000134247	–1.70007	2.23E-75	PTGFRN
ENSG00000154864	–2.43789	1.97E-68	PIEZO2
ENSG00000137809	–2.88538	2.49E-67	ITGA11
ENSG00000198691	–2.87645	2.77E-67	ABCA4
ENSG00000132561	–4.16833	5.78E-67	MATN2
ENSG00000164692	–2.66029	2.88E-64	COL1A2
ENSG00000090006	–1.81295	1.33E-62	LTBP4
2B			
ENSG00000183117	9.562538	2.79E-11	CSMD1
ENSG00000241945	7.372619287	1.03E-30	PWP2
ENSG00000179750	7.162458435	1.80E-08	APOBEC3B
ENSG00000160221	6.924046677	6.72E-31	GATD3A
ENSG00000265185	6.806274092	0.001104238	SNORD3B-1
ENSG00000167619	6.706105751	0.000661441	TMEM145
ENSG00000275708	6.182372504	0.000919537	MIR3648-1
ENSG00000274611	5.791504304	0.016067411	TBC1D3
ENSG00000130540	5.539785314	0.000658834	SULT4A1
ENSG00000145936	5.487773143	0.000567221	KCNMB1
ENSG00000274419	5.439096586	0.00102837	TBC1D3D
ENSG00000159648	5.423595889	6.87E-05	TEPP
ENSG00000226864	5.352608919	0.001976085	ATE1-AS1
ENSG00000275895	5.101613111	3.65E-10	U2AF1L5
ENSG00000114812	5.098758045	0.019157021	VIPR1
ENSG00000198300	5.051708848	0.00070514	PEG3
ENSG00000178222	5.026864484	2.04E-12	RNF212
ENSG00000275713	5.015806533	0.018162042	HIST1H2BH
ENSG00000101746	4.917796086	0.017228264	NOL4
ENSG00000198889	4.915680791	3.83E-07	DCAF12L1
2C			
ENSG00000134762	–9.232296422	0.033718361	DSC3
ENSG00000103175	–7.955204006	6.57E-05	WFDC1
ENSG00000274512	–7.926806431	2.00E-11	TBC1D3
ENSG00000046604	–7.814993348	4.09E-09	DSG2
ENSG00000164100	–7.792194728	1.74E-06	NDST3
ENSG00000127324	–7.699937782	2.74E-11	TSPAN8
ENSG00000162706	–7.475371727	4.69E-78	CADM3
ENSG00000276345	–7.233761932	6.18E-10	LOC107987373
ENSG00000136011	–7.065863539	2.65E-07	STAB2
ENSG00000139865	–6.708376838	0.001509194	TTC6
ENSG00000153976	–6.641890017	0.003354519	HS3ST3A1
ENSG00000213088	–6.603983188	0.000116847	ACKR1
ENSG00000122176	–6.587131149	1.36E-53	FMOD
ENSG00000144648	–6.458119012	4.56E-05	ACKR2
ENSG00000127507	–6.40028912	0.000166137	ADGRE2
ENSG00000198910	–6.386203743	7.70E-09	L1CAM
ENSG00000157766	–6.320851197	5.63E-05	ACAN
ENSG00000189431	–6.287886418	0.000482488	RASSF10
ENSG00000118849	–6.204673965	6.05E-07	RARRES1
ENSG00000065609	–6.170758791	0.000115977	SNAP91

When data were analysed by fc regardless of significance level, 172 DEGs were up-regulated ≥2 fc, of these 38 were up-regulated with a ≥4 fc. The top five most up-regulated genes were *CSMD1, PWP2, APOBEC3B, GATD3A,* and *SNORD3B-1* all of which were significant *p* ≤ 0.001, Table 2B. Of the DEGs in HPMEC, 398 were down-regulated ≤ –2 fc with 91 down-regulated with a ≤ –4 fc. The top five most down-regulated genes, all of which were significant *p* ≤ 0.001, were *DSC3, WFDC1, TBC1D3, DSG2,* and *NDST3*; see Table 2C.

### Pathway analysis of DEGs in HPMECs

KEGG and Reactome databases within EnrichR were used for pathway analysis of DEGs in HPMEC between PAH and controls. Using the DEGs in HPMEC that were significant *p* ≤ 0.0001, KEGG analysis showed involvement of a number of relevant pathways, Supplement Table 1A. Of interest are ECM–receptor interaction (*p* = 3.414e-11), focal adhesion (*p* = 4.189e-9), PI3K-Akt signalling pathway (*p* = 1.809e-7), cell adhesion molecules (CAMs) (*p* = 1.701e-7), platelet activation (*p* = 0.000127), protein digestion and absorption (*p* = 0.00032). All these pathways, with exception of platelet activation, were still highlighted and significant (*p* ≤ 0.0001) when only DEGs with *p* ≤ 0.000001 were used. Most of the genes related to these pathways were down-regulated in PAH-HPMEC. Using the Reactome database for analysis of the same DEGs in HPMEC, similar pathways were highlighted with the top pathway again being ECM organisation, (*p* = 1.962e-26), Supplement Table 1B. In addition, Cytokine Signalling in immune system (*p* = 3.636e-7) was identified. When only the DEGs with ≥2 fc were analysed, the Reactome database revealed involvement of the following pathways: interferon alpha/beta signalling (*p* = 6.025e-15), interferon signalling (*p* = 2.524e-9), Cytokine Signalling in immune system (*p* = 0.0000023), chemokine receptors bind chemokines (*p* = 0.000607), immune system (*p* = 0.01237), collagen degradation (*p* = 0.01237) and Interferon gamma signalling (*p* = 0.007831); Supplement Table 2A. Analysis using genes with ≥4 Log2 fc showed a weak association with collagen degradation (*p* = 0.1271), activation of MMPs (*p* = 0.1271), senescence-associated secretory phenotype (*p* = 0.2021) and degradation of the ECM (*p* = 0.2021).

The same analysis but using KEGG database showed very few pathways to be significant, Supplement Table 2B. Of interest were cytokine–cytokine receptor interaction (*p* = 0.00106), Toll-like receptor signalling pathway (*p* = 0.03512) and chemokine signalling pathway (*p* = 0.2524). Using genes with ≥4 Log2 fc showed a weak association with aldosterone synthesis and secretion (*p* = 0.3405).

Using DEGs with ≤ –2 fc, both KEGG and Reactome analysis showed similar pathways to be important to those seen with the significantly regulated genes (Table 3). In addition, the transforming growth factor (TGF)-β signalling pathway (*p* = 0.02164) was highlighted.

**Table 3.** The top 10 pathways highlighted by KEGG (A) and Reactome (B) analysis of the down-regulated (≤ –2 Log2 fold change) DEGs in human pulmonary microvascular endothelial cells from PAH patients compared to controls ranked by combined score

Index	Name	*p*-Value	Adjusted *p*-value	Z-score	Combined score
A: KEGG analysis.					
1	ECM-receptor interaction_Homo sapiens_hsa04512	1.948e-15	3.253e-13	–1.71	58.01
2	Focal adhesion_Homo sapiens_hsa04510	2.426e-8	0.0000020	–1.90	33.26
3	PI3K-Akt signaling pathway_Homo sapiens_hsa04151	3.962e-7	0.0000166	–1.99	29.32
4	Protein digestion and absorption_Homo sapiens_hsa04974	2.212e-7	0.0000123	–1.66	25.44
5	Amoebiasis_Homo sapiens_hsa05146	0.00003	0.0008525	–1.73	18.02
6	Cell adhesion molecules (CAMs)_Homo sapiens_hsa04514	0.000028	0.0008525	–1.63	17.09
7	Hippo signaling pathway_Homo sapiens_hsa04390	0.000058	0.001383	–1.59	15.53
8	Arrhythmogenic right ventricular cardiomyopathy (ARVC)_Homo sapiens_hsa05412	0.00011	0.002292	–1.58	14.39
9	Platelet activation_Homo sapiens_hsa04611	0.00076	0.01400	–1.71	12.26
10	TGF-beta signaling pathway_Homo sapiens_hsa04350	0.001423	0.02164	–1.57	10.30
B: Reactome analysis					
1	Extracellular matrix organization_Homo sapiens_R-HSA-1474244	5.459e-13	3.144e-10	–2.11	59.60
2	Collagen formation_Homo sapiens_R-HSA-1474290	1.184e-10	3.410e-8	–2.02	46.16
3	Collagen biosynthesis and modifying enzymes_Homo sapiens_R-HSA-1650814	2.687e-10	5.158e-8	–2.00	44.13
4	Diseases of glycosylation_Homo sapiens_R-HSA-3781865	2.056e-9	2.961e-7	–1.87	37.41
5	Assembly of collagen fibrils and other multimeric structures_Homo sapiens_R-HSA-2022090	7.503e-9	8.644e-7	–1.99	37.23
6	Glycosaminoglycan metabolism_Homo sapiens_R-HSA-1630316	1.854e-8	0.000002	–1.91	33.94
7	Axon guidance_Homo sapiens_R-HSA-422475	5.533e-7	0.000040	–2.31	33.32
8	Diseases associated with glycosaminoglycan metabolism_Homo sapiens_R-HSA-3560782	5.564e-7	0.000040	–1.94	27.90
9	Developmental Biology_Homo sapiens_R-HSA-1266738	0.0000082	0.00048	–2.30	27.00
10	ECM proteoglycans_Homo sapiens_R-HSA-3000178	0.0000013	0.000081	–1.80	24.42

In summary, PAH-HPMEC display significant alterations (down-regulation) in pathways involved in ECM-receptor organisation, CAMs and focal adhesion signalling together with up-regulation of cytokine and chemokine pathways, collagen degradation and activation of MMPs.

### Ontology analysis of DEGs in HPMECs

Biological processes analysis of the DEGs in HPMEC which were significant (*p* ≤ 0.0001 and *p* ≤ 0.000001) revealed involvement of ECM organisation, cell-matrix adhesion, cytokine-mediated signalling pathway and Type I interferon signalling pathway, Supplement Table 3A. Using DEGs with ≥2 fc, Type 1 interferon signalling and cellular response to interferon appear to be important biological processes in HPMEC, Supplement Table 3B. When DEGs, which were down-regulated ≤ –2 fc, were examined, ECM organisation was highly significant, Supplement Table 3C.

Gene ontology (GO) Molecular Function analysis of significant (*p* ≤ 0.0001 and *p* ≤ 0.000001) DEGs in HPMEC suggested that platelet-derived growth factor binding (*p* = 0.00165) and scavenger receptor activity (*p* = 0.00139) could be important. Using only up-regulated genes with ≥2 fc analysis showed CXCR chemokine receptor binding, cytokine receptor binding and chemokine activity to be important in HPMEC. When genes with ≤ –2 fc were used, similar Molecular Functions to the analysis of significantly regulated genes was seen.

GO cellular component analysis of HPMEC using DEGs, which were significant (*p* ≤ 0.0001 and *p* ≤ 0.000001), showed involvement of endoplasmic reticulum lumen, lysosomal lumen, vacuolar lumen, focal adhesion and platelet alpha granule. Considering only DEGs in HPMEC with ≥2 fc, no Cellular Components were significant. However, when genes with ≤ –2 fc were used, significance was seen in relation to the plasma membrane, endoplasmic reticulum lumen and platelet dense granule.

In summary, PAH-HPMEC exhibited up-regulation of processes related to chemokine and cytokine receptor binding and chemokine activity with down-regulation of ECM organisation. These processes could be linked to down-regulation of components of the endoplasmic reticulum, lysosome, and vacuole lumens.

### Analysis of transcription factors linked to DEGs in PAH-HPMECs

Two databases were used for this analysis: Transfac & Jaspar PWMs and Transcription Factor PPI. Using Transfac & Jaspar analysis of significant (*p* ≤ 0.0001 and *p* ≤ 0.000001) DEGs in HPMEC showed ZNF148, SP3, KLF11, TEAD2 and KLF4 to be the top five significant (*p* < 0.05) transcription factors, Supplement Table 4A. Using only the up-regulated DEGs with ≥ 2 fc, analysis showed no transcription factors to be significant. When DEGs with ≤–2 Log2 fc were used, KLF11 and KLF4 were highlighted as significant (*p* < 0.002) with ARNT and ZNF148 also significant (*p* < 0.02) and TEAD2 almost reaching significance, *p* < 0.07, Supplement Table 4B. When the same DEGs were analysed against Transcription Factor PPI database, different transcription factors were highlighted as being important, although not as significant. The top two were ATF2 and RAD21 (*p* < 0.02). When only the up-regulated genes with ≥2 Log2 fc or the down-regulated genes with ≤–2 Log2 fc were used, no significant involvement of transcription factors was shown.

**Table 4.** The top 10 most (A) significant (B) up-regulated (≥ 1.5 Log2 fold change) (C) down-regulated (≤–1.5 Log2 fold change) microRNAs in human pulmonary microvascular endothelial cells from PAH patients compared to controls.

	log2 fold change	padj	Symbol
4A			
ENSG00000207807	–4.577	9.81E-59	MIR95
ENSG00000283170	–3.211	2.41E-37	MIR382
ENSG00000276365	–5.301	2.86E-37	MIR145
ENSG00000194717	–4.178	3.69E-33	MIR494
ENSG00000207749	–3.827	3.30E-31	MIR299
ENSG00000212040	–3.326	1.16E-30	MIR543
ENSG00000207754	–3.218	8.71E-29	MIR487B
ENSG00000202560	–3.203	3.15E-27	MIR539
ENSG00000207743	–3.460	9.18E-26	MIR495
ENSG00000198982	–2.983	9.18E-26	MIR380
4B			
ENSG00000283242	29.994	0.00557	MIR384
ENSG00000261122	29.732	7.0E-08	LINC02167
ENSG00000207349	5.047	0.01848	RNVU1-17
ENSG00000266104	4.944	0.00029	MIR4326
ENSG00000266852	4.780	0.00125	MIR4482
ENSG00000265390	3.263	0.02557	MIR4999
ENSG00000239183	2.485	0.04969	SNORA84
ENSG00000264773	2.332	0.01748	MIR4420
ENSG00000207813	2.319	0.00086	MIR605
ENSG00000211459	1.666	0.00117	RNR1
4C			
ENSG00000264725	–5.306	5.69E-20	MIR3129
ENSG00000276365	–5.301	2.86E-37	MIR145
ENSG00000230937	–5.252	0.025568	MIR205HG
ENSG00000202270	–4.778	0.002658	SNORD114-12
ENSG00000221525	–4.762	1.31E-11	MIR1185-1
ENSG00000283588	–4.699	2.13E-06	MIR376A1
ENSG00000207807	–4.577	9.81E-59	MIR95
ENSG00000207938	–4.364	1.57E-19	MIR511
ENSG00000275662	–4.249	0.007672	SNORD112
ENSG00000194717	–4.178	3.69E-33	MIR494

In summary, the transcription factors KLF4, KLF11, ZNF148 and TEAD2 appear to be of possible importance in PAH-HPMEC.

### Differentially expressed microRNAs in PAH-HPMEC compared to controls

One-hundred and six microRNAs in HPMEC were significantly (*p* ≤ 0.01) up- or down-regulated in PAH compared to controls with 49 out of the top 50 being down-regulated. The five most significantly changed microRNAs in HPMEC were *miR-95, miR-382, miR-145, miR-494, miR-299*; Table 4A. Analysis by fc, regardless of significance level, showed that only 10 differentially expressed microRNAs were up-regulated ≥ 1.5 fc, of these six were also significant, *p* ≤ 0.01. The top five most up-regulated microRNAs in HPMEC were found to be *miR-384, LINCO2167, RNVU1-17, miR-4326, miR-4482,* all of which were significant at *p* ≤ 0.05, Table 4B. A total of 94 microRNAs were down-regulated ≤ –1.5 fc in HPMEC with 17 out of the top 20 being significant *p* ≤ 0.01. The top five most down-regulated microRNAs in HPMEC were found to be *miR-3129, miR-145, miR-205HG, SNORD114-12, and miR-1185-1*; Table 4C. Of note, *miR-145* was the third most significant and the second most down-regulated microRNA in PAH-HPMEC.

### Pathway analysis of differentially expressed microRNAs in HPMECs

Using KEGG and Reactome databases within EnrichR for analysis of differentially expressed microRNAs that were significant at *p* ≤ 0.01 or up-regulated ≥ 1.5 fc showed no pathways to be important. Reactome analysis of the microRNAs which were down-regulated ≤ 1.5 fc suggested (*p* < 0.09) Pre-NOTCH Transcription and Translation and Pre-NOTCH Expression and Processing pathways to be of possible importance in PAH-HPMEC.

### Ontology analysis of differentially expressed microRNAs in HPMECs

Analysis of differentially expressed microRNAs either by significance or by up- or down-regulation showed no Biological Processes, Molecular Functions or Cellular Components to be of importance in PAH-HPMEC.

### Analysis of transcription factors linked to differentially expressed microRNAs in HPMECs

Analysis using Transfac & Jaspar PWMs or Transcription Factor PPI of the differentially expressed microRNAs in PAH-HPMEC either by significance or by up- or down-regulation showed no transcription factors to be important.

### DEGs in PAH-HPASMCs compared to controls

In HPASMC, there were 229 DEGs between PAH and controls which were significant (*p* ≤ 0.0001) and of these 162 genes were still significant at *p* ≤ 0.000001. Out of the top 30 significant genes, 16 were up-regulated and 14 down-regulated. The top five most significantly regulated genes in HPASMC were *ISLR* (–2.2 fc)*, SCD* (3.1 fc), *DHCR24* (2.6 fc)*, PAPPA2* (–2.7 fc) and *DMKN* (2.2 fc); see Table 5A. HPASMC displayed 257 DEGs which were up-regulated ≥ 2 fc in PAH and of these 71 were up-regulated with a ≥ 4 fc. The top five most up-regulated genes were found to be *XIST, PSG4, ADGRF5, CDH5* and *PECAM1*, all of which were significant *p* ≤ 0.001, Table 5B. Of the DEGs in HPASMC, 232 were discovered to be down-regulated ≤ –2 fc with 58 down-regulated ≤ –4 fc. The top five most down-regulated genes, all of which were significant *p* ≤ 0.0005, were *DDX3Y, RPS4Y1, ZFY, NLGN4Y* and *PRKY,* see Table 5C.

**Table 5.** The top 20 (A) significant (B) up-regulated and (C) down-regulated DEGs in human pulmonary artery smooth muscle cells from PAH patients compared to controls.

	log2 fold change	padj	Symbol
5A			
ENSG00000129009	–2.25668	8.03E-159	ISLR
ENSG00000099194	3.123026	2.64E-132	SCD
ENSG00000116133	2.635486	1.33E-128	DHCR24
ENSG00000116183	–2.78162	6.22E-125	PAPPA2
ENSG00000161249	2.289074	5.01E-118	DMKN
ENSG00000185633	–3.12061	1.32E-113	NDUFA4L2
ENSG00000203875	–2.16321	1.31E-99	SNHG5
ENSG00000118971	–2.17407	8.16E-97	CCND2
ENSG00000168477	–2.21771	5.64E-91	TNXB
ENSG00000163975	–2.21607	3.83E-77	MELTF
ENSG00000133048	6.177547	3.23E-76	CHI3L1
ENSG00000138829	2.347783	2.44E-70	FBN2
ENSG00000134824	2.238076	6.77E-70	FADS2
ENSG00000128918	5.423211	4.47E-69	ALDH1A2
ENSG00000175899	3.022507	7.24E-69	A2M
ENSG00000189223	–2.74364	8.50E-62	PAX8-AS1
ENSG00000243137	8.371158	6.41E-57	PSG4
ENSG00000168079	–3.60722	9.62E-56	SCARA5
ENSG00000152580	2.758684	3.11E-51	IGSF10
ENSG00000188153	3.394363	2.89E-49	COL4A5
5B			
ENSG00000229807	10.12355	1.68E-28	XIST
ENSG00000243137	8.371158	6.41E-57	PSG4
ENSG00000069122	8.226087	2.90E-06	ADGRF5
ENSG00000179776	8.141403	9.78E-10	CDH5
ENSG00000261371	7.155848	1.71E-14	PECAM1
ENSG00000164669	7.01257	2.35E-06	INTS4P1
ENSG00000128917	7.007891	0.004991	DLL4
ENSG00000162706	6.84867	8.96E-11	CADM3
ENSG00000189398	6.586894	2.92E-06	OR7E12P
ENSG00000251226	6.571353	3.82E-05	LOC729305
ENSG00000125810	6.539587	1.82E-08	CD93
ENSG00000133048	6.177547	3.23E-76	CHI3L1
ENSG00000184005	5.992962	9.28E-06	ST6GALNAC3
ENSG00000163219	5.913127	1.69E-05	ARHGAP25
ENSG00000189420	5.672965	0.00037	ZFP92
ENSG00000162711	5.586885	0.009927	NLRP3
ENSG00000223756	5.566061	1.04E-30	TSSC2
ENSG00000091879	5.494023	4.48E-05	ANGPT2
ENSG00000204287	5.475197	0.003675	HLA-DRA
ENSG00000130035	5.452035	0.028274	KCNA6
5C			
ENSG00000067048	–11.3908	9.28E-21	DDX3Y
ENSG00000129824	–10.47	3.03E-29	RPS4Y1
ENSG00000067646	–10.1682	7.13E-12	ZFY
ENSG00000165246	–9.80645	1.60E-11	NLGN4Y
ENSG00000099725	–9.65163	9.03E-11	PRKY
ENSG00000233864	–9.63986	1.47E-10	TTTY15
ENSG00000114374	–9.50301	1.35E-10	USP9Y
ENSG00000198692	–9.22846	2.26E-10	EIF1AY
ENSG00000131002	–9.20194	5.98E-10	TXLNGY
ENSG00000012817	–8.04687	1.92E-15	KDM5D
ENSG00000183878	–7.40202	2.86E-06	UTY
ENSG00000104435	–6.98774	6.06E-22	STMN2
ENSG00000196436	–6.81156	1.89E-07	NPIPB15
ENSG00000101307	–6.74899	2.29E-13	SIRPB1
ENSG00000171798	–6.54804	5.52E-16	KNDC1
ENSG00000241106	–6.54328	0.000172	HLA-DOB
ENSG00000176165	–6.54061	1.19E-11	FOXG1
ENSG00000277586	–6.12609	2.02E-18	NEFL
ENSG00000255346	–6.02201	0.000225	NOX5
ENSG00000184809	–5.93371	0.00053	B3GALT5-AS1

### Pathway analysis of DEGs in HPASMCs

KEGG analysis of significantly (*p* ≤ 0.0001) regulated DEGs in HPASMC revealed fatty acid metabolism (*p* = 0.1706), biosynthesis of unsaturated fatty acids (*p* = 0.1706) and CAMs (*p* = 0.3049) pathways to be important, Supplement Table 5A. When analysis was performed using only the genes with *p* ≤ 0.000001, the same pathways were highlighted but with greater significance. Using the Reactome database for analysis of the same DEGs in HPASMC, similar pathways were highlighted, Supplement Table 5B.

When the DEGs in HPASMC with ≥ 2 fc change were analysed using KEGG, similar pathways were highlighted to those seen when analysis was performed using the significantly expressed genes; namely, the Fatty acid and CAM pathways, but these were significant at *p* ≤ 0.01, see Table 6A. Likewise, using Reactome database for the same analysis of genes with ≥ 2 fc, the same fatty acid and ECM organisation pathways were highlighted, Table 6B. When genes with ≥ 4 fc were used, Reactome identified cell junction, cell–cell junctions, adherens junctions and cell communication appear to be important. Using DEGs in HPASMC with either a ≤ –2 or ≤ –4 fc, both KEGG and Reactome analysis showed no significant pathways to be altered.

**Table 6.** List of the top 10 pathways highlighted by KEGG (A) and Reactome (B) analysis of the up-regulated (≥ 2 Log2 fold change) DEGs in human pulmonary artery smooth muscle cells from PAH patients compared to controls ranked by combined score.

Index	Name	*p*-Value	Adjusted *p*-Value	Z-score	Combined score
6A: KEGG analysis					
1	Cell adhesion molecules (CAMs)_Homo sapiens_hsa04514	0.00000230	0.0003687	–1.73	22.40
2	Fatty acid metabolism_Homo sapiens_hsa01212	0.00003315	0.002652	–1.83	18.93
3	Malaria_Homo sapiens_hsa05144	0.0004050	0.01620	–1.82	14.24
4	Biosynthesis of unsaturated fatty acids_Homo sapiens_hsa01040	0.0001946	0.01038	–1.51	12.88
5	PI3K-Akt signaling pathway_Homo sapiens_hsa04151	0.004798	0.1279	–1.94	10.34
6	Focal adhesion_Homo sapiens_hsa04510	0.004708	0.1279	–1.82	9.76
7	Inflammatory bowel disease (IBD)_Homo sapiens_hsa05321	0.009771	0.2233	–1.74	8.06
8	Ras signaling pathway_Homo sapiens_hsa04014	0.02778	0.3594	–1.75	6.27
9	Leukocyte transendothelial migration_Homo sapiens_hsa04670	0.01824	0.3594	–1.55	6.19
10	ECM-receptor interaction_Homo sapiens_hsa04512	0.02134	0.3594	–1.47	5.65
6B: Reactome analysis					
1	Kinesins_Homo sapiens_R-HSA-983189	0.00001991	0.009098	–1.97	21.29
2	Extracellular matrix organization_Homo sapiens_R-HSA-1474244	0.00008089	0.01706	–2.10	19.82
3	Axon guidance_Homo sapiens_R-HSA-422475	0.0003839	0.04022	–2.34	18.40
4	Linoleic acid (LA) metabolism_Homo sapiens_R-HSA-2046105	0.0001120	0.01706	–1.64	14.95
5	alpha-linolenic (omega3) and linoleic (omega6) acid metabolism_Homo sapiens_R-HSA-2046104	0.0005453	0.04022	–1.95	14.63
6	alpha-linolenic acid (ALA) metabolism_Homo sapiens_R-HSA-2046106	0.0005453	0.04022	–1.92	14.44
7	COPI-dependent Golgi-to-ER retrograde traffic_Homo sapiens_R-HSA-6811434	0.0006161	0.04022	–1.88	13.87
8	Signaling by VEGF_Homo sapiens_R-HSA-194138	0.003592	0.1252	–2.41	13.57
9	Generation of second messenger molecules_Homo sapiens_R-HSA-202433	0.001020	0.05824	–1.94	13.36
10	Hemostasis_Homo sapiens_R-HSA-109582	0.002184	0.1074	–2.08	12.77

In summary, PAH-HPASMC show up-regulation of fatty acid biosynthesis and metabolism pathways together with an increase in cell adhesion, cell–cell junctions and communication pathways.

### Ontology analysis of DEGs in HPASMCs

Analysis of GO Biological Processes using DEGs in HPASMC that were significant (*p* ≤ 0.0001 and *p* ≤ 0.000001) showed intermediate filament bundle assembly (*p* = 0.1978) and regulation of positive chemotaxis (*p* = 0.2217) could be relevant processes. When up-regulated genes with ≥ 2 fc were analysed, the Biological Processes related to Regulation of positive chemotaxis and Fatty acid metabolic processes were highlighted. Analysis of genes with ≤ –2 fc suggested that the biological process of intermediate filament bundle assembly could be important in PAH-HPASMC.

Using, GO Molecular Function analysis of HPASMC using DEGs that were significant (*p* ≤ 0.0001 and *p* ≤ 0.000001), none were found to be significant. However, using DEGs with ≥2 fc analysis revealed the possible involvement of microtubule motor activity (*p* = 0.003579), ATPase activity (*p* = 0.05302) and motor activity (*p* = 0.01678). No significant Molecular Functions were established when genes with ≤–2 fc were used.

GO cellular component analysis of HPASMC using DEGs which were significant (*p* ≤ 0.0001 and *p* ≤ 0.000001) showed involvement of the integral and anchored components of the plasma membrane. Using only the up-regulated genes ≥2 fc analysis displayed changes in Kinesin complex, condensed chromosome kinetochore, integral component of plasma membrane and polymeric cytoskeletal fibre. With DEGs down-regulated ≤ –2 fc in HPASMC analysis revealed involvement of the integral and anchored components of the plasma membrane to be important.

In summary, PAH-HPASMC showed up-regulation of processes related to positive chemotaxis and fatty acid metabolism with increased microtubule motor activity and kinesin complexes potentially driving a hyperproliferative cell phenotype.

### Analysis of transcription factors linked to DEGs in HPASMCs

When analysis was performed on DEGs in HPASMC that were significant (*p* ≤ 0.0001 and *p* ≤ 0.000001), Transfac & Jaspar showed TFAP2A (*p* = 0.08) to be the only transcription factor of potential importance. When genes in HPASMC were analysed by fc, no significant transcription factors were shown. Similarly, when Transcription Factor PPI database was used for the analysis whether at the significance level or fc, no involvement of transcription factors was indicated.

In summary, no transcription factors were shown to be significant in PAH-HPASMC.

### Differentially expressed microRNAs in PAH-HPASMCs compared to controls

There were 27 microRNAs in HPASMC which are either significantly (*p* < 0.01) up- or down-regulated in PAH compared to controls, with 11 microRNAs being up-regulated and 16 down-regulated. The five most significantly changed microRNAs in HPASMC were, *SNORA74B, miR-126, miR-532, miR-362 and miR-4326*; see Table 7A. HPASMC displayed 10 differentially expressed microRNAs which were up-regulated ≥1.5 fc with 6 out of the 10 also being significant *p* ≤ 0.01. The top five most up-regulated microRNAs in HPASMC were found to be *miR-153-1, miR-615, miR-187, miR-126 and miR-6892*, all of which were significant *p* ≤ 0.05, Table 7B. Of the differentially expressed microRNAs, 11 were down-regulated ≤ –1.5 fc. Of these 11 microRNAs, 8 were significant at *p* < 0.01. The top five most down-regulated microRNAs were *miR-4326, miR-346, LOC100506688, SNORA74B and miR-4443*, all significant *p* ≤ 0.05, Table 7C. Of interest are two microRNAs: *SNORA74B* which was the most significantly expressed and the fourth most down-regulated and *miR-126* which was the second most significant and the fourth most up-regulated microRNA in HPASMC. *miR-4326* appeared to be important in both PAH cell types; it was the fourth most up-regulated microRNA in HPMEC but was the most down-regulated and fifth most significant microRNA in HPASMC.

**Table 7.** The top 10 most (A) significant (B) up-regulated (C) down-regulated microRNAs in human pulmonary artery smooth muscle cells from PAH patients compared to controls ranked by fold change.

	log2 fold change	padj	Symbol
7A
ENSG00000212402	–3.73435	5.07E-32	SNORA74B
ENSG00000199161	2.105047	5.38E-15	MIR126
ENSG00000207758	–1.82158	1.55E-11	MIR532
ENSG00000208015	–1.38364	2.88E-08	MIR362
ENSG00000266104	–6.47494	1.05E-06	MIR4326
ENSG00000199104	–5.8899	1.73E-06	MIR346
ENSG00000207571	2.374125	1.41E-05	MIR615
ENSG00000207970	–1.31096	3.68E-05	MIR660
ENSG00000207932	1.683621	5.92E-05	MIR33A
ENSG00000264661	–0.99827	0.000217	MIR3200
7B
ENSG00000207647	2.582343	0.000291	MIR153-1
ENSG00000207571	2.374125	1.41E-05	MIR615
ENSG00000207797	2.161331	0.002252	MIR187
ENSG00000199161	2.105047	5.38E-15	MIR126
ENSG00000278449	1.99872	0.026522	MIR6892
ENSG00000283160	1.946334	0.001474	MIR4521
ENSG00000283468	1.821494	0.022249	MIR4454
ENSG00000207932	1.683621	5.92E-05	MIR33A
ENSG00000199059	1.563962	0.001602	MIR135B
ENSG00000264720	1.535385	0.035781	MIR3117
7C
ENSG00000266104	–6.47494	1.05E-06	MIR4326
ENSG00000199104	–5.8899	1.73E-06	MIR346
ENSG00000215246	–5.74964	0.01043	LOC100506688
ENSG00000212402	–3.73435	5.07E-32	SNORA74B
ENSG00000265483	–2.87173	0.001463	MIR4443
ENSG00000207717	–2.38897	0.035781	MIR551B
ENSG00000211513	–1.97061	0.009825	MIR320E
ENSG00000207758	–1.82158	1.55E-11	MIR532
ENSG00000207785	–1.75393	0.001976	MIR500A
ENSG00000272080	–1.69684	0.006214	MIR502

### Pathway analysis of differentially expressed microRNAs in HPASMCs

KEGG analysis of differentially expressed microRNAs that were significant *p* ≤ 0.01 or up- or down-regulated ≥ 1.5 fc showed no pathways to be important. Reactome analysis of the microRNAs which were significant *p* ≤ 0.01 suggested Pre-NOTCH Transcription and Translation and Pre-NOTCH Expression and Processing pathways (*p* = 0.008) and MAPK6/MAPK4 signalling (*p* = 0.014) to be of possible importance in PAH-HPMEC. Analysis using up or down-regulated microRNAs showed no pathways to be important.

### Ontology analysis of differentially expressed microRNAs in HPASMCs

Analysis of differentially expressed microRNAs either by significance or by up- or down-regulation showed no Biological Processes, Molecular Functions or Cellular Components to be of importance in PAH-HPASMC.

### Analysis of transcription factors linked to differentially expressed microRNAs in HPASMCs

Analysis using Transfac & Jaspar PWMs or Transcription Factor PPI of the differentially expressed microRNAs in PAH-HPASMC either by significance or by up- or down-regulation showed no transcription factors to be important.

### Validation of DEGs in each cell type using qRT-PCR

qRT-PCR was used to validate six of the top genes (*DSC3, DSG2, TSPAN8, CADM3, STAB2, TTC6*) which were down-regulated in HPMEC (Supplement Figure 1A) and six of the top up-regulated genes (*ADGRF5, CDH5, PECAM1, INTS4P1, CADM3, LOC729305*) in HPASMC (Supplement Figure 1B). Data obtained from these RT-PCR experiments in each cell type confirmed the RNA-Sequence data.

**Fig. 1.**
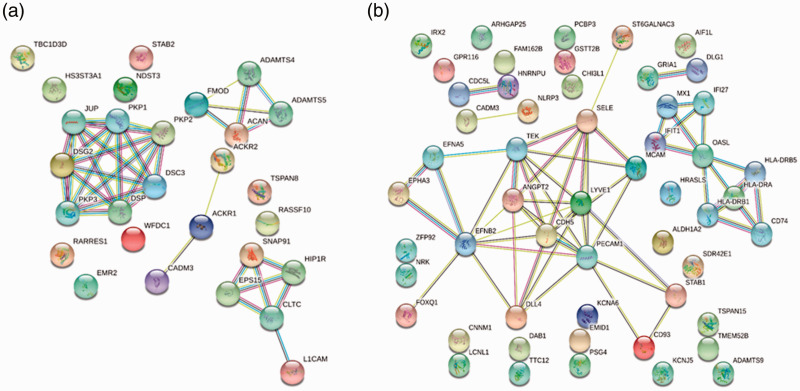
String diagrams of the protein–protein interactions in (a) pulmonary microvascular endothelial cells using the 20 most down-regulated DEGs and (b) pulmonary artery smooth muscle cells using the top 50 up-regulated DEGs in PAH patients compared to controls. *Nodes*: Network nodes represent proteins, splice isoforms or post-translational modifications are collapsed, i.e. each node represents all the proteins produced by a single, protein-coding gene locus. *Coloured nodes*: Query proteins and first shell of interactors. *White nodes*: Second shell of interactors. *Empty nodes*: Proteins of unknown 3D structure. *Filled nodes*: Some 3D structure is known or predicted. *Edges*: Edges represent protein–protein associations. Associations are meant to be specific and meaningful, i.e. proteins jointly contribute to a shared function; this does not necessarily mean they are physically binding each other. *Known interactions*: From curated databases. Experimentally determined. *Predicted interactions*: Gene neighbourhood, Gene fusions, Gene co-occurrence. *Others*: Text mining, co-expression, protein homology.

The potential protein–protein interactions for the DEGs in each vascular cell type are summarised in String diagrams, Fig. 1.

## Discussion

In this study, we utilised RNA and microRNA sequence analysis of primary pulmonary microvascular ECs and pulmonary artery smooth muscle cells from patients with PAH and non-PAH controls. We demonstrated differences in gene expression at baseline between PAH and controls within each cell type. At baseline, PAH-HPMEC displayed twice as many down-regulated genes compared to up-regulated, but in PAH-PASMC almost equal numbers of genes were up- or down-regulated. The top five most significantly changed DEGs in PAH-HPMEC, all of which were down-regulated, were *PKHD1L1, ITGA1, POSTN, RELN* and *COL4A5*. In the same cells, the five most down-regulated genes were *DSC3, WFDC1, TBC1D3, DSG2* and *NDST3*. Pathway analysis of down-regulated genes (≤ –2 fc) showed these genes were linked to ECM organisation, collagen formation/biosynthesis and cell and focal adhesion pathways. The top five most up-regulated DEGs were *CSMD1, PWP2, APOBEC3B, GATD3A* and *SNORD3B-*1. Pathway analysis of the up-regulated genes showed Cytokine Signalling and interactions, collagen degradation and activation of MMPs to be most important. The five most significantly regulated DEGs in PAH-HPASMC were *ISLR, SCD, DHCR24, PAPPA2* and *DMKN.* The top five most up-regulated genes were found to be *XIST, PSG4, ADGRF5, CDH5* and *PECAM1*. Pathway analysis of these up-regulated DEGs showed biosynthesis of unsaturated fatty acids and fatty acid metabolism to be important. The top five most down-regulated genes were *DDX3Y, RPS4Y1, ZFY, NLGN4Y* and *PRKY*.

Gene expression studies using microarrays and RNA sequencing have been performed in animal models of PAH but only a few human studies on PAH-derived samples. Human studies have been performed on lung homogenates giving a global view of PAH^22^ or on laser capture microdissection samples^23^ giving a more compartment specific view or on circulating cell expression profiles.^24^ Cell type-specific studies using isolated primary cells from PAH patients are very limited. In a recent study, Rhodes et al. used RNA sequencing for the first time to investigate the transcriptome of human healthy and PAH ECs. The authors proposed a link between BMPR2 dysfunction and the reduced expression of endothelial collagen IV (COL4) and ephrin A1 (EFNA1) which may initiate vulnerability of the endothelium to injury in PAH.^25^ COL4 is one of the main components of the EC basement membrane and EFNA1 is an EC guidance molecule^26^ that induces the release of COL4 from ECs.^27^ In the same study, a loss in BMPR2 signalling in ECs impaired their ability to produce COL4 and EFNA1 by a mechanism which involved a decrease in β-catenin, a known downstream effector of BMPR2 gene regulation.^28^ Cell culture models using siRNA demonstrated that decreases in these proteins caused negative effects on EC adhesion and migration and in transgenic mice lacking EFNA1, a greater loss of distal arteries was seen associated with a worsening of PAH severity. The same authors also showed a decrease in both IQSEC1 (IQ motif and Sec7 domain 1), a guanidine nucleotide exchange protein which is recruited by epidermal growth factor receptor / vascular endothelial growth factor receptor 2 (EGFR/VEGFR2) and interferon-stimulated exonuclease gene 20kD (ISG20). IQSEC1 stimulates angiogenesis and controls the turnover of β-integrins^29^ making it important in cell adhesion. In human umbilical vein, EC dominant-negative ISG20 inhibited angiogenic function.

In agreement with the above study, our RNA-sequence data, which was performed to a greater depth of reads, a limitation noted in the data of Rhodes et al., also showed down regulation of collagen proteins in ECs. COL4A5 was the fifth most significantly down-regulated DEG in PAH-HPMEC and also COL4A6 was significantly down-regulated by –2.5 fc. In total, 17 different collagen mRNAs were significantly (*p* < 0.05) down-regulated and only 2 (COL15A1 and COL18A1) were significantly up-regulated. Additionally, EFNA1 and CTNNB1 (β-catenin) were also significantly down-regulated in PAH compared to non-PAH controls adding support to the findings of Rhodes et al. IQSEC1 was down-regulated but in contrast ISG20 was up-regulated in PAH-HPMEC in our study. Of interest in PAH-PASMC, few changes in collagen were seen; COL4A5 and COL4A6 and COL21A1 were significantly up-regulated and down-regulation was only seen in COL10A1 and COL11A2.

Rhodes et al linked their findings to a reduction in BMPR2 signalling in PAH-HPMEC. Our data did not show any differences in BMPR2 signalling between PAH and controls in either cell type. Although not investigated specifically, this suggests that our patients did not possess BMPR2 mutations. However, significant down-regulation of BMPR1A and BMPR1B was seen in PAH-HPMEC together with a decrease in BMP1 and 4. No differences in BMP receptors were seen in PAH-PASMC but an increase in BMP2 was seen. This suggests that down regulation of collagen synthesis in pulmonary microvascular ECs may occur independent of BMPR2 signalling and needs to be investigated further.

In our study, PAH-HPMEC demonstrate major dysregulation of the ECM and basement membrane, possibly leading to loss of cell–matrix and cell–cell interactions. EC dysfunction is thought to be an early event in PAH progression. Increased vascular stiffness and increased pulsatile flow due to remodelling of the ECM of the pulmonary arteries is thought to play a critical role in the pathogenesis of PAH and by some to be a cause rather than a consequence of distal microvascular proliferative vasculopathy.^30^ Composition of the ECM is regulated by the balance between proteolytic enzymes such as the MMPs, metalloproteases (ADAMs), lysyl oxidases (LOXs) and serine elastases and their endogenous regulators, TIMPs. An imbalance in these proteolytic enzymes and their inhibitors has been reported in PAH linked to an increase in collagen deposition.^31^ Pulmonary arteries from PAH patients show altered expression of several MMPs, ADAMs, LOXs, serine elastases and TIMPs in the intima and media layers compared to healthy controls.^17,32,33^ Our data show dysregulation of these proteolytic enzymes and inhibitors in PAH-HPMEC but not in PAH-PASMC. PAH-HPMEC showed significant up-regulation of metalloproteinases MMP1, MMP7, MMP10, MMP15 and MMP28 together with down-regulation of MMP2, MMP16 and MMP19. There was a significant down-regulation of ADAM and ADAMTS proteases, namely ADAM19 and ADAM33, ADAMTS2, 10, 12, 15 and ADAMTSL3. Of note ADAM11, ADAMTS4, 6, 9, 13 and 18 were significantly up-regulated; however, the adjusted *p* values for these DEGs were much higher. Significant down-regulation of the lysyl oxidase LOXL1 was seen in PAH-HPMEC together with significant down-regulation of TIMP1 and TIMP3. Down-regulation of the hyaluronan synthases (HAS1 and HAS3) was seen in both cell types; however, only PAH-HPMEC showed an increase in hyaluronidases (HYAL2 and HYAL3). Hyaluronidases degrade hyaluronan, one of the major glycosaminoglycans of the ECM and up-regulation in HPMEC may add to the endothelial dysfunction.

It is still unclear what mechanism(s) drive the imbalance in these proteolytic enzymes in the pulmonary arteries of patients with PAH. Several have been proposed but alterations in the function and structure of ECs have been forwarded as the initiating events. Increases in shear stress, flow, pulsatility and inflammation can all lead to pulmonary EC damage, loss of barrier function and increased permeability. These changes allow PASMC to encounter various known and “unidentified” factors which cause the PASMC to secrete elastases.^15^ Serine elastases can degrade the ECM allowing activation/release of growth factors and signalling molecules from the ECM such as TGF-β which can stimulate PASMC and fibroblasts to proliferate and also produce ECM components, e.g. collagen and elastin.^34^ Furthermore, growth factors and ECM breakdown products can also lead to an increase in MMPs.

Initiation of the imbalance in proteolytic enzymes and their inhibitors, which result in pulmonary vascular ECM remodelling, has been linked to inflammation.^35^ Inflammation in PAH patients and animal models of PAH is proposed as a major driver of pulmonary vascular remodelling.^36–38^ Increases in proteolytic enzymes leads to an increase in proinflammatory breakdown products of the ECM, which results in a positive feedback loop.

Accumulating evidence suggests that endothelial to mesenchymal transition (EndMT) could be an important link between inflammatory stress and endothelial dysfunction. During EndMT, ECs undergo a phenotypic switch whereby they start to lose their endothelial characteristics and develop a mesenchymal phenotype showing increased expression of genes with a similar profile to smooth muscle cells.^39^ In the process of EndMT, ECs separate from the intimal monolayer due to loss of cell–ECM and cell–cell interactions resulting from down-regulation of endothelial gene markers such as vascular endothelial (VE)-cadherin, desmoplakin and cytokeratins. ECs are then able to migrate into the medial layer and become myofibroblast-like mesenchymal cells which display increased expression of α-smooth muscle actin, vimentin, BMP4 and MMPs.^39^ Numerous stimuli implicated in the pathogenesis of PAH can initiate EndMT. Inflammatory cytokines IL-1β, IL-6, TNFα, transcription factor NF-κB, endotoxin and ROS have all been shown to cause EndMT mostly via activation of the TGF-β signalling pathway.

Our data revealed significant down-regulation of EC markers such as desmoplakin and four different cytokeratins (KRT7, 8, 18 and 80) and up-regulation of mesenchymal markers α-SMA, BMP4 and TGF-β2 in PAH-HPMEC cells compared to controls. Interestingly, our data also showed a significant up-regulation of EC markers VE-cadherin and PECAM-1 in PAH-PASMC compared to controls. We could speculate that some of the EC are partially transitioned cells undergoing EndMT and that some of the PASMC are actually fully transitioned EC which have undergone EndMT but keep a mixed phenotype suggesting an ‘identity crisis’.^40^ Suzuki et al. recently demonstrated the presence of fully transitioned and partial transitioned EndMT in a PAH model using endothelial-specific lineage mice. Partial transitioned cells in this model were enriched for EPC markers such as CD133 and CD34 which were lost in fully transitioned cells.^41^ Indeed, our data showed that CD34 was significantly up-regulated in PAH-HPMEC compared to controls. Of importance our data has highlighted ‘*what is actually a true pulmonary endothelial cell or pulmonary artery smooth muscle cell in PAH*’? Since both cell types express markers of the other cell type something which is not seen in controls, it appears that these two cell types in disease have lost their true phenotype showing plasticity with characteristics of both.

Krüppel-like factors (KLFs) are integral regulators of vascular homeostasis. KLF4 expression is decreased during turbulent flow due to methylation within the KLF4 promoter by DNA methyltransferase.^42^ Down-regulation of KLF2/4 due to disturbed flow leads to up-regulation of NF-κB, a major driver of inflammatory processes.^43,44^ Additionally KLF11 is highly expressed in ECs and suppresses inflammation via direct binding to the p65 subunit of NF-κB. Microarray analysis of lung tissue from patients with PAH demonstrated that KLF4 gene expression was decreased^45^ and Q-PCR and Western blot analysis by another group also confirmed that KLF4 was decreased in PAH lung tissue.^46^

Analysis of the DEGs in PAH-HPMEC in our study confirmed that the transcription factors KLF4 and KLF11 could be important in the pathogenesis of PAH. Both KLF4 and KLF11 were each linked to the down-regulation of 54 DEGs in PAH-HPMEC. Whether the actual levels of these transcription factors are altered or if their activity is changed needs to be determined. KLFs can be regulated by post-transcriptional regulation for example by microRNAs.

miR-145 is highly expressed in vascular smooth muscle cells and is a critical regulator of the contractile or differentiated phenotype of these cells and dysregulation of this miRNA is connected to vascular injury^47^ and may contribute to the development of PAH.^48^ Down-regulation of miR-145 is associated with intimal hyperplasia possibly via ETS-like gene 1.^47^ Decreases in this miRNA leads to an increase in angiotensin 1-converting enzyme and angiotensin II (AngII).^47^ AngII is a potent activator of NF-κB pathway and increases blood pressure within the vasculature by causing vessels to constrict.^49^ EC/PASMC interactions induce the activation of miR-145 transcription in PASMC, which promotes the transfer of this miRNA to ECs via membrane nanotubes or tunnelling nanotubes.^50^ A mechanism proposed is that secretion of TGF-β by ECs stimulates the transfer. Our data showed no difference in miR-145 in PASMC; however, there was significant down-regulation of this miRNA in PAH-HPMEC. Perhaps one could speculate that this transfer process is dysregulated in PAH leading to down-regulation of miR-145 in ECs resulting in a pro-inflammatory and pro-thrombotic environment within the endothelial layer, which could promote intima thickening and lesion formation within the pulmonary vasculature.

In contrast, PASMCs from patients with PAH did not show down-regulation/dysfunction of ECM remodelling but instead showed up-regulation of fatty acid biosynthesis.

In normal healthy cells, energy is derived mainly from glucose oxidation. Glucose is converted to pyruvate, which can enter the mitochondria and Krebs cycle via the enzyme pyruvate dehydrogenase (PDH) instead of remaining in the cytoplasm and undergoing glycolysis. In PAH, PDH is inhibited and glucose oxidation via the mitochondria is prevented with pyruvate entering other anaplerotic reactions and amino acid biosynthesis which are crucial processes for proliferating cells. Energy production still occurs in the mitochondria but there is a switch from using pyruvate to the use of fatty acids as a fuel source. A result of increased energy metabolism is a shift from mitochondrial oxidative phosphorylation (OXPHOS) to increased glycolysis or ‘Warburg Effect’.^51^ This metabolic process is a potential driver of apoptosis resistance and mitochondrial dysfunction.^52,53^ Proliferating cells such as PAH-HPASMC have a higher rate of de novo fatty acid synthesis to provide lipids for cell membranes and energy requirements.^54^ In a mouse model of PAH, inhibition of fatty acid oxidation by inhibiting malonyl CoA decarboxylase suppressed the development of PAH by decreasing cytoplasmic glycolysis and increasing mitochondrial-based OXPHOS together with suppression of HIF1α activation.^55,56^ In addition, free fatty acids can modulate essential processes, which drive cell survival such as apoptosis and autophagy.^57,58^ Arachidonic acid metabolites are elevated in PAH patients and play a role in the contractile response and prevent apoptosis. They can also initiate and propagate cell proliferation via their effects on signalling pathways, mitogens and cell cycle.^57^ This suggests that inhibition of fatty acid synthesis/oxidation could be a potential therapeutic approach to treat PAH and warrants further investigation.

There are limitations to this work. Our PAH patient samples were not genotyped or phenotyped, so we do not know if any carried known mutations related to PAH. This was a small study involving only four donors per group and the two vascular cell types were not isolated from the same donors. Vascular cells from PAH patients were collected at end-stage disease (transplant or time of death), thus reflect severe PAH. Both these aspects are due to the technical difficulties of using primary human derived samples. Ideally, it would be interesting to look at different stages of PAH progression to map disease but there is no way of obtaining cells from the pulmonary microvasculature whilst a donor is alive. Additionally, there are no animal models that recapitulate all aspects of human PAH. Finally, these data are only observational, and hypothesis generating and must be investigated/validated in other larger PAH cohorts. Cell culture studies need to be performed to explore potential mechanisms in this patient group.

Diagnosis of PAH is still problematic as patients experience a broad range of symptoms and the earlier stages of onset are mainly asymptomatic. There is need for improvement in the early detection of PAH as patients diagnosed in functional classes 1 and 2 show better survival rates than patients diagnosed in higher functional class disease.

It is still unclear if initial damage to the pulmonary vasculature activates an inflammatory response or whether inflammation (toxin/pathogen-mediated) damages the vasculature in the early stages of disease development. Many of these early metabolic changes have been shown to occur in the pulmonary vasculature long before pathophysiologic changes making early disease detection a challenge. Much work is still required to try and identify the initial stages of PAH so disease can be targeted much earlier.

In summary, analysis of the two key structural cells involved in PAH show distinct patterns of gene expression, HPMECs displayed over four times as many DEGs between PAH and control samples than HPASMCs. There was a significant down-regulation of ECM and cell–cell interaction pathways in HPMECs, whilst there was evidence for an enhanced energy-driven proliferative phenotype in HPASMCs. More interestingly, we observed a blurring of the differences between the two cell types in disease with each possessing features considered specific for the other. Future studies should utilise single-cell RNA sequencing to determine whether this reflects distinct cell activation states or cell types. The identification of distinct mechanistic roles implicated in HPMECs and HPASMCs function in PAH pathogenesis and the interactions between these cells should be confirmed in ex vivo artery-on-a-chip studies using selective pathway modulators. These studies will also indicate whether the cell-specific pathways shown here are the initiators or consequences of PAH.

## Supplemental Material

sj-pdf-1-pul-10.1177_2045894021996190 - Supplemental material for Extracellular matrix degradation pathways and fatty acid metabolism regulate distinct pulmonary vascular cell types in pulmonary arterial hypertension Click here for additional data file. Supplemental material, sj-pdf-1-pul-10.1177_2045894021996190 for Extracellular matrix degradation pathways and fatty acid metabolism regulate distinct pulmonary vascular cell types in pulmonary arterial hypertension by Sharon Mumby, F. Perros, C. Hui, B.L. Xu, W. Xu, V Elyasigomari, A. Hautefort, G. Manaud, M. Humbert, K.F. Chung, S.J. Wort and I.M. Adcock in Pulmonary Circulation

sj-pdf-2-pul-10.1177_2045894021996190 - Supplemental material for Extracellular matrix degradation pathways and fatty acid metabolism regulate distinct pulmonary vascular cell types in pulmonary arterial hypertension Click here for additional data file. Supplemental material, sj-pdf-2-pul-10.1177_2045894021996190 for Extracellular matrix degradation pathways and fatty acid metabolism regulate distinct pulmonary vascular cell types in pulmonary arterial hypertension by Sharon Mumby, F. Perros, C. Hui, B.L. Xu, W. Xu, V Elyasigomari, A. Hautefort, G. Manaud, M. Humbert, K.F. Chung, S.J. Wort and I.M. Adcock in Pulmonary Circulation

sj-pdf-3-pul-10.1177_2045894021996190 - Supplemental material for Extracellular matrix degradation pathways and fatty acid metabolism regulate distinct pulmonary vascular cell types in pulmonary arterial hypertension Click here for additional data file. Supplemental material, sj-pdf-3-pul-10.1177_2045894021996190 for Extracellular matrix degradation pathways and fatty acid metabolism regulate distinct pulmonary vascular cell types in pulmonary arterial hypertension by Sharon Mumby, F. Perros, C. Hui, B.L. Xu, W. Xu, V Elyasigomari, A. Hautefort, G. Manaud, M. Humbert, K.F. Chung, S.J. Wort and I.M. Adcock in Pulmonary Circulation

sj-pdf-4-pul-10.1177_2045894021996190 - Supplemental material for Extracellular matrix degradation pathways and fatty acid metabolism regulate distinct pulmonary vascular cell types in pulmonary arterial hypertension Click here for additional data file. Supplemental material, sj-pdf-4-pul-10.1177_2045894021996190 for Extracellular matrix degradation pathways and fatty acid metabolism regulate distinct pulmonary vascular cell types in pulmonary arterial hypertension by Sharon Mumby, F. Perros, C. Hui, B.L. Xu, W. Xu, V Elyasigomari, A. Hautefort, G. Manaud, M. Humbert, K.F. Chung, S.J. Wort and I.M. Adcock in Pulmonary Circulation
