# Supplementary figures and images for: Extracellular matrix degradation pathways and fatty acid metabolism regulate distinct pulmonary vascular cell types in pulmonary arterial hypertension
Source: Pulm Circ. 2021 Mar 2;11(1):2045894021996190. doi: 10.1177/2045894021996190 (PMC8366141; doi:10.1177/2045894021996190)

1B

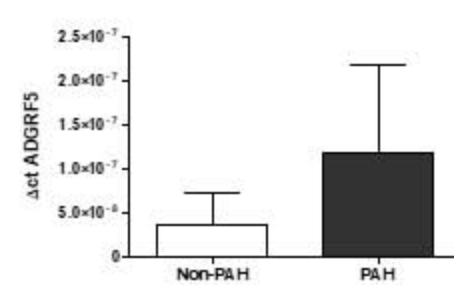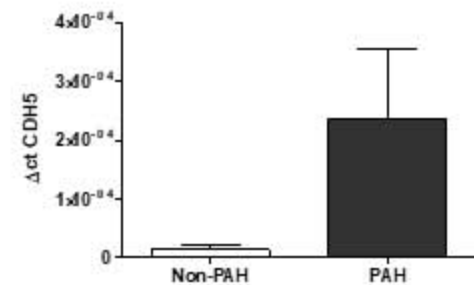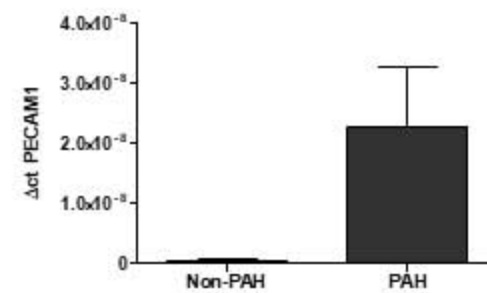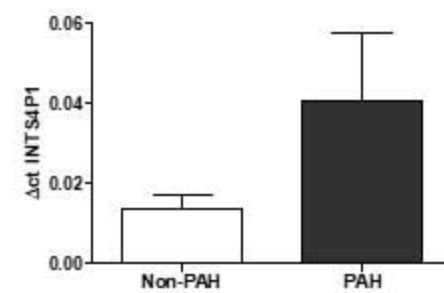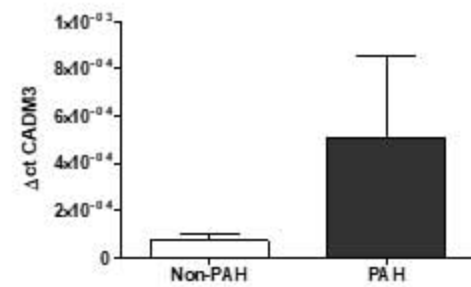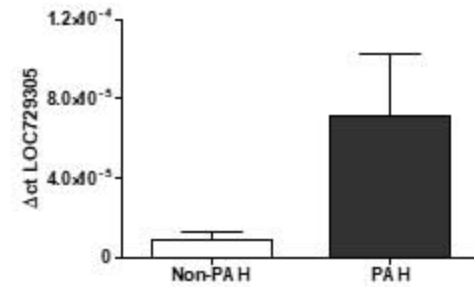

Supplement: sj-pdf-1-pul-10.1177_2045894021996190 - Supplemental material for Extracellular matrix degradation pathways and fatty acid metabolism regulate distinct pulmonary vascular cell types in pulmonary arterial hypertension [file sj-pdf-1-pul-10.1177_2045894021996190.pdf]

1B

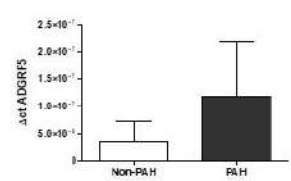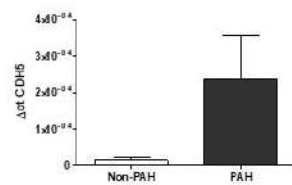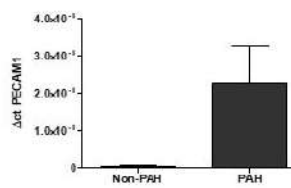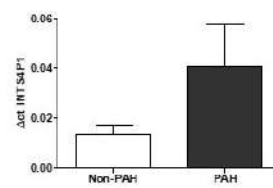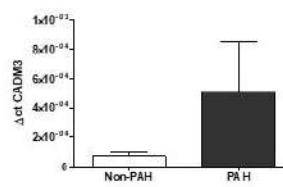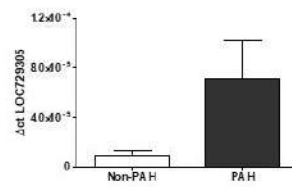

Supplement: sj-pdf-2-pul-10.1177_2045894021996190 - Supplemental material for Extracellular matrix degradation pathways and fatty acid metabolism regulate distinct pulmonary vascular cell types in pulmonary arterial hypertension [file sj-pdf-2-pul-10.1177_2045894021996190.pdf]

1A

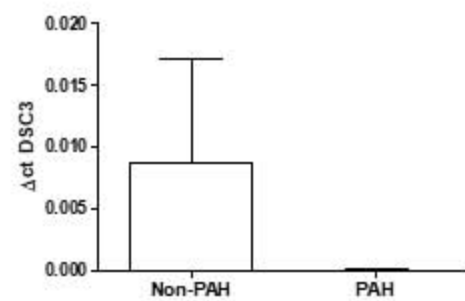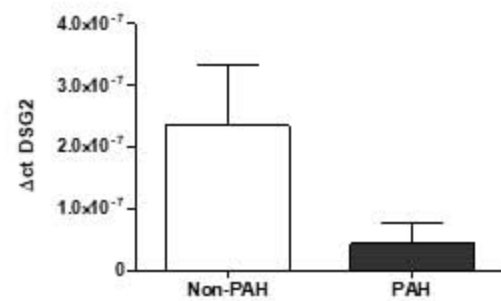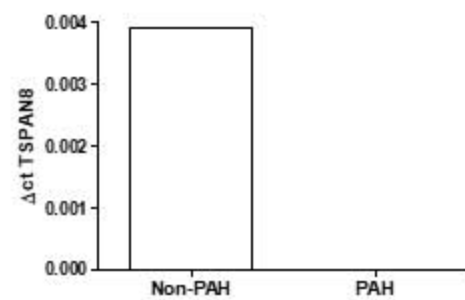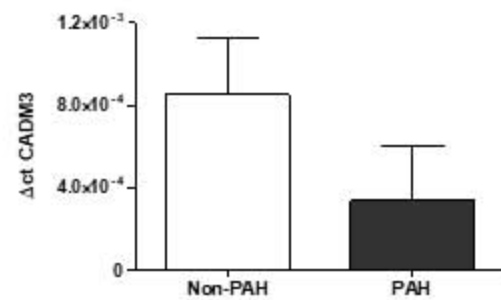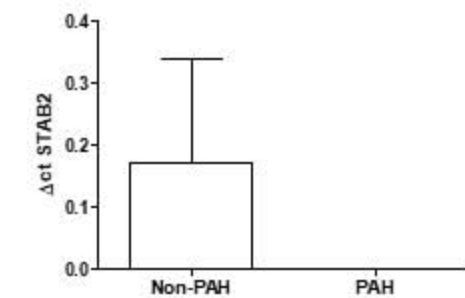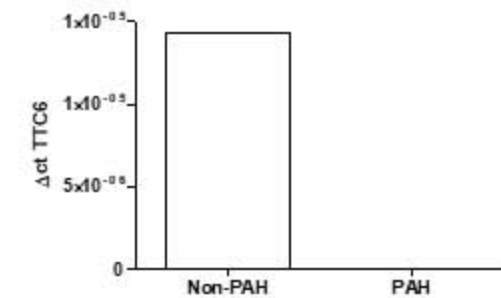

Supplement: sj-pdf-3-pul-10.1177_2045894021996190 - Supplemental material for Extracellular matrix degradation pathways and fatty acid metabolism regulate distinct pulmonary vascular cell types in pulmonary arterial hypertension [file sj-pdf-3-pul-10.1177_2045894021996190.pdf]
