# Supplementary material for: Extracellular matrix degradation pathways and fatty acid metabolism regulate distinct pulmonary vascular cell types in pulmonary arterial hypertension
Source: Pulm Circ. 2021 Mar 2;11(1):2045894021996190. doi: 10.1177/2045894021996190 (PMC8366141; doi:10.1177/2045894021996190)
Supplement: sj-pdf-4-pul-10.1177_2045894021996190 - Supplemental material for Extracellular matrix degradation pathways and fatty acid metabolism regulate distinct pulmonary vascular cell types in pulmonary arterial hypertension [file sj-pdf-4-pul-10.1177_2045894021996190.pdf]

Supplement Table 1: List of the top 10 pathways highlighted by KEGG (A) and Reactome (B) analysis of the significant (p<0.0001) DEGs in human pulmonary microvascular endothelial cells from PAH patients compared to controls ranked by combined score.

S1A=KEGG analysis

Index	Name	P-value	Adjusted p-value	Z-score	Combined score
1	ECM-receptor interaction_Homo sapiens_hsa04512	1.308e-13	3.414e-11	-1.71	50.80
2	Focal adhesion_Homo sapiens_hsa04510	3.210e-11	4.189e-9	-1.90	45.83
3	PI3K-Akt signaling pathway_Homo sapiens_hsa04151	3.465e-9	1.809e-7	-1.96	38.25
4	Regulation of actin cytoskeleton_Homo sapiens_hsa04810	2.607e-9	1.701e-7	-1.85	36.50
5	Cell adhesion molecules (CAMs)_Homo sapiens_hsa04514	2.130e-9	1.701e-7	-1.68	33.50
6	Platelet activation_Homo sapiens_hsa04611	0.000002939	0.0001278	-1.78	22.73
7	Amoebiasis_Homo sapiens_hsa05146	0.000009735	0.0003203	-1.71	19.68
8	Protein digestion and absorption_Homo sapiens_hsa04974	0.000009818	0.0003203	-1.51	17.42
9	Arrhythmogenic right ventricular cardiomyopathy (ARVC)_Homo sapiens_hsa05412	0.00001676	0.0004862	-1.54	16.98
10	Phagosome_Homo sapiens_hsa04145	0.00003149	0.0008219	-1.49	15.45

S1B=Reactome analysis

Index	Name	P-value	Adjusted p-value	Z-score	Combined score
1	Extracellular matrix organization_Homo sapiens_R-HSA-1474244	1.726e-29	1.962e-26	-2.11	139.80
2	Axon guidance_Homo sapiens_R-HSA-422475	3.235e-14	1.839e-11	-2.36	73.20
3	Collagen formation_Homo sapiens_R-HSA-1474290	2.595e-12	9.837e-10	-2.01	53.63
4	Developmental Biology_Homo sapiens_R-HSA-1266738	2.895e-10	6.584e-8	-2.35	51.59
5	Assembly of collagen fibrils and other multimeric structures_Homo sapiens_R-HSA-2022090	6.560e-11	1.865e-8	-2.00	46.94
6	Cytokine Signaling in Immune system_Homo sapiens_R-HSA-1280215	3.198e-9	3.636e-7	-2.32	45.47
7	Hemostasis_Homo sapiens_R-HSA-109582	4.336e-10	8.216e-8	-2.11	45.45
8	Platelet activation, signaling and aggregation_Homo sapiens_R-HSA-76002	1.572e-9	2.234e-7	-2.10	42.65
9	Collagen biosynthesis and modifying enzymes_Homo sapiens_R-HSA-1650814	1.139e-9	1.850e-7	-1.96	40.37
10	Diseases of glycosylation_Homo sapiens_R-HSA-3781865	1.820e-9	2.299e-7	-1.83	36.76

Supplement Table 2: List of the top 10 pathways highlighted by Reactome (A) and KEGG (B) analysis of the up-regulated (≥ 2 log2 fold change) DEGS in human pulmonary microvascular endothelial cells from PAH patients compared to controls ranked by combined score.

S2A=Reactome analysis

**Index**	**Name**	**P-value**	**Adjusted p-value**	**Z-score**	**Combined score**
1	Interferon alpha/beta signaling_Homo sapiens_R-HSA-909733	1.674e-17	6.025e-15	-1.87	72.04
2	Interferon Signaling_Homo sapiens_R-HSA-913531	1.402e-11	2.524e-9	-2.09	52.34
3	Cytokine Signaling in Immune system_Homo sapiens_R-HSA-1280215	1.918e-8	0.000002301	-2.39	42.41
4	Peptide ligand-binding receptors_Homo sapiens_R-HSA-375276	0.000006862	0.0006070	-1.95	23.13
5	Chemokine receptors bind chemokines_Homo sapiens_R-HSA-380108	0.000008430	0.0006070	-1.92	22.45
6	Class A/1 (Rhodopsin-like receptors)_Homo sapiens_R-HSA-373076	0.0001154	0.006922	-2.10	19.05
7	G alpha (i) signalling events_Homo sapiens_R-HSA-418594	0.0002395	0.01078	-2.11	17.59
8	Immune System_Homo sapiens_R-HSA-168256	0.0003276	0.01237	-2.16	17.37
9	Collagen degradation_Homo sapiens_R-HSA-1442490	0.0003435	0.01237	-2.03	16.18
10	Interferon gamma signaling_Homo sapiens_R-HSA-877300	0.0001523	0.007831	-1.74	15.25

S2B=KEGG analysis

**Index**	**Name**	**P-value**	**Adjusted p-value**	**Z-score**	**Combined score**
1	Influenza A_Homo sapiens_hsa05164	0.000002871	0.0003158	-1.97	25.13
2	Cytokine-cytokine receptor interaction_Homo sapiens_hsa04060	0.00001931	0.001062	-1.85	20.12
3	RIG-I-like receptor signaling pathway_Homo sapiens_hsa04622	0.0003420	0.01254	-1.67	13.29
4	Hepatitis C_Homo sapiens_hsa05160	0.001033	0.02125	-1.76	12.11
5	Measles_Homo sapiens_hsa05162	0.001159	0.02125	-1.73	11.71
6	Rheumatoid arthritis_Homo sapiens_hsa05323	0.001082	0.02125	-1.66	11.34
7	Toll-like receptor signaling pathway_Homo sapiens_hsa04620	0.002235	0.03512	-1.74	10.62
8	Herpes simplex infection_Homo sapiens_hsa05168	0.005369	0.07383	-1.61	8.42
9	Epithelial cell signaling in Helicobacter pylori infection_Homo sapiens_hsa05120	0.02083	0.2524	-1.66	6.44
10	Chemokine signaling pathway_Homo sapiens_hsa04062	0.02295	0.2524	-1.69	6.40

Supplement Table 3: List of the top 10 processes highlighted by GO Biological processes analysis of the **(A)** significant ($p \leq 0.0001$), **(B)** ≥ 2 Log2 fold change and **(C)** ≤ -2 Log2 fold change DEGs in human pulmonary microvascular endothelial cells from PAH patients compared to controls ranked by combined score

S3A

Index	Name	P-value	Adjusted p-value	Z-score	Combined score
1	extracellular matrix organization (GO:0030198)	1.921e-23	6.589e-20	-1.65	86.12
2	regulated exocytosis (GO:0045055)	1.109e-7	0.0000634	-2.30	36.78
3	positive regulation of ion transmembrane transporter activity (GO:0032414)	0.000001	0.0003809	-2.48	33.58
4	glycosaminoglycan catabolic process (GO:0006027)	9.325e-8	0.0000634	-1.94	31.46
5	cytokine-mediated signaling pathway (GO:0019221)	8.773e-11	1.504e-7	-1.34	31.14
6	embryonic eye morphogenesis (GO:0048048)	0.000012	0.001859	-2.65	30.10
7	regulation of cellular response to growth factor stimulus (GO:0090287)	0.000024	0.002885	-2.81	29.91
8	type I interferon signaling pathway (GO:0060337)	0.000004	0.001015	-2.30	28.56
9	collagen biosynthetic process (GO:0032964)	0.00020	0.01195	-3.02	25.71
10	regulation of single stranded viral RNA replication via double stranded DNA intermediate (GO:0045091)	0.00012	0.008596	-2.65	24.03

S3B

Index	Name	P-value	Adjusted p-value	Z-score	Combined score
1	type I interferon signaling pathway (GO:0060337)	1.028e-17	4.680e-15	-2.33	90.96
2	cellular response to type I interferon (GO:0071357)	1.028e-17	4.680e-15	-1.40	54.57
3	negative regulation of viral genome replication (GO:0045071)	1.318e-14	3.003e-12	-1.47	47.05
4	regulation of nuclease activity (GO:0032069)	1.812e-7	0.00002358	-2.85	44.23
5	regulation of viral genome replication (GO:0045069)	7.989e-15	2.426e-12	-1.35	43.88
6	negative regulation of viral life cycle (GO:1903901)	1.653e-13	2.510e-11	-1.42	41.80
7	cytokine-mediated signaling pathway (GO:0019221)	1.609e-13	2.510e-11	-1.34	39.53
8	response to type I interferon (GO:0034340)	0.00003391	0.002207	-2.98	30.66
9	regulation of tyrosine phosphorylation of STAT protein (GO:0042509)	0.00001530	0.001267	-2.16	23.94
10	regulation of transposition (GO:0010528)	0.0004638	0.02010	-2.50	19.16

S3C

Index	Name	P-value	Adjusted p-value	Z-score	Combined score
1	extracellular matrix organization (GO:0030198)	1.848e-14	3.701e-11	-1.65	52.07
2	bundle of His cell to Purkinje myocyte communication (GO:0086069)	0.0006009	0.03196	-3.60	26.72
3	glycosaminoglycan catabolic process (GO:0006027)	0.000001729	0.0007690	-1.94	25.79
4	glycosaminoglycan biosynthetic process (GO:0006024)	1.138e-8	0.00001140	-1.27	23.29
5	positive regulation of urine volume (GO:0035810)	0.0004066	0.03196	-2.87	22.43
6	desmosome organization (GO:0002934)	0.0006009	0.03196	-2.96	21.96
7	skeletal system development (GO:0001501)	0.000001472	0.0007690	-1.47	19.77
8	cell-matrix adhesion (GO:0007160)	0.000001920	0.0007690	-1.49	19.57

Index	Name	P-value	Adjusted p-value	Z-score	Combined score
9	neuron projection extension involved in neuron projection guidance (GO:1902284)	0.0006009	0.03196	-2.51	18.62
10	aorta development (GO:0035904)	0.0006009	0.03196	-2.49	18.49

Supplement Table 4: List of the top 10 Transcription factors highlighted by Transfac & Jaspar analysis of the **(A)** significant ($p \leq 0.0001$), **(B)** ≤ -2 Log2 fold change DEGs in human pulmonary microvascular endothelial cells from PAH patients compared to controls ranked by combined score.

S4A

Index	Name	P-value	Adjusted p-value	Z-score	Combined score
1	ZNF148 (human)	0.000002813	0.0008384	-1.74	22.19
2	SP3 (human)	0.00001369	0.002040	-1.75	19.59
3	KLF11 (human)	0.00004460	0.004431	-1.73	17.30
4	TEAD2 (human)	0.00006733	0.005016	-1.70	16.37
5	KLF4 (human)	0.0001023	0.005827	-1.67	15.35
6	ZBTB7A (human)	0.0001173	0.005827	-1.66	15.06
7	TCFAP2A (human)	0.0001563	0.006653	-1.69	14.79
8	REPIN1 (human)	0.0003479	0.01296	-1.68	13.35
9	PCBP1 (human)	0.0004747	0.01572	-1.66	12.69
10	SP1 (mouse)	0.0007796	0.02323	-1.52	10.91

S4B

Index	Name	P-value	Adjusted p-value	Z-score	Combined score
1	KLF11 (human)	0.000001694	0.0004692	-1.75	23.29
2	KLF4 (human)	0.00001210	0.001676	-1.71	19.36
3	ARNT (human)	0.0002218	0.01885	-1.78	15.00
4	ZNF148 (human)	0.0002722	0.01885	-1.70	13.94
5	TEAD2 (mouse)	0.001497	0.06913	-1.67	10.86
6	SMAD4 (mouse)	0.001296	0.06913	-1.63	10.85
7	ZBTB7A (human)	0.003681	0.1456	-1.65	9.26
8	NFATC2 (human)	0.02962	0.8204	-1.89	6.66
9	TFAP2A (mouse)	0.02164	0.7493	-1.60	6.12
10	REPIN1 (human)	0.02632	0.8100	-1.66	6.05

Supplement Table 5: List of the top 10 pathways highlighted by KEGG (A) and Reactome (B) analysis of the significant (p≤0.0001) DEGs in human pulmonary artery smooth muscle cells from PAH patients compared to controls ranked by combined score.

S5A=KEGG analysis

Index	Name	P-value	Adjusted p-value	Z-score	Combined score
1	Fatty acid metabolism_Homo sapiens_hsa01212	0.002195	0.1706	-1.88	11.52
2	Biosynthesis of unsaturated fatty acids_Homo sapiens_hsa01040	0.002215	0.1706	-1.59	9.71
3	Cell adhesion molecules (CAMs)_Homo sapiens_hsa04514	0.005940	0.3049	-1.68	8.60
4	PI3K-Akt signaling pathway_Homo sapiens_hsa04151	0.01710	0.4074	-1.96	7.99
5	Hypertrophic cardiomyopathy (HCM)_Homo sapiens_hsa05410	0.01522	0.4074	-1.75	7.33
6	Dilated cardiomyopathy_Homo sapiens_hsa05414	0.01990	0.4074	-1.75	6.84
7	Wnt signaling pathway_Homo sapiens_hsa04310	0.02388	0.4074	-1.70	6.36
8	Protein digestion and absorption_Homo sapiens_hsa04974	0.01990	0.4074	-1.54	6.03
9	Adrenergic signaling in cardiomyocytes_Homo sapiens_hsa04261	0.02791	0.4074	-1.66	5.94
10	Focal adhesion_Homo sapiens_hsa04510	0.02910	0.4074	-1.67	5.91

S5B=Reactome analysis

Index	Name	P-value	Adjusted p-value	Z-score	Combined score
1	Extracellular matrix organization_Homo sapiens_R-HSA-1474244	0.0004405	0.1722	-2.11	16.31
2	EPHA-mediated growth cone collapse_Homo sapiens_R-HSA-3928663	0.006825	0.3786	-2.36	11.76
3	Signaling by VEGF_Homo sapiens_R-HSA-194138	0.01361	0.3786	-2.45	10.52
4	Signaling by Interleukins_Homo sapiens_R-HSA-449147	0.01525	0.3786	-2.41	10.08
5	Linoleic acid (LA) metabolism_Homo sapiens_R-HSA-2046105	0.003493	0.3414	-1.70	9.63
6	VEGFR2 mediated cell proliferation_Homo sapiens_R-HSA-5218921	0.02447	0.3786	-2.45	9.08
7	alpha-linolenic (omega3) and linoleic (omega6) acid metabolism_Homo sapiens_R-HSA-2046104	0.009369	0.3786	-1.91	8.91
8	alpha-linolenic acid (ALA) metabolism_Homo sapiens_R-HSA-2046106	0.009369	0.3786	-1.88	8.79
9	Signaling by Retinoic Acid_Homo sapiens_R-HSA-5362517	0.01225	0.3786	-1.93	8.48
10	VEGF binds to VEGFR leading to receptor dimerization_Homo sapiens_R-HSA-195399	0.003493	0.3414	-1.45	8.22
